# Accidental bufotoxin intoxication: Arenobufagin identification by liquid chromatography coupled to mass spectrometry

**DOI:** 10.1016/j.plabm.2024.e00424

**Published:** 2024-08-15

**Authors:** Alessandro Bonari, Mauro Leucio Mattei, Giovanni Cappelli, Francesca Romano, Nicoletta Cini, Francesca Luceri, Donato Squillaci, Stefano Dugheri, Alessandra Fanelli, Nicola Mucci

**Affiliations:** a General Laboratory, Azienda Ospedaliero-Universitaria Careggi, Florence, Italy; b Department of Experimental and Clinical Medicine, University of Florence, 50121, Florence, Italy

**Keywords:** Bufotoxin, Liquid chromatography, Mass spectrometry, Pharmacotoxicology

## Abstract

Background: Since ancient times, poisoning, even serious poisoning, has been known to occur during nature walks. Intentional or unintentional ingestion of toxins of animal origin is one of the possible causes of poisoning. Bufadienolide poisoning is a critical case. This is because of its high potency and its ability to cross the blood-brain barrier. Due to the rarity of these poisonings in humans in Central Europe, their identification is often difficult. The following is a case report of a poisoning by toad eggs in an Italina child, that presented vertigo, fussiness and sleepiness. A method of toxin identification using the prince of pharmacotoxicology, liquid chromatography (LC) coupled with tandem mass spectrometry (MS/MS), and an innovative reasoning were used. This method can be applied to other poisoning cases.

## 1 Introduction

Poisoning, even serious poisoning, has been known to occur in nature since ancient times [1]. One of the possible causes of poisoning is the ingestion, whether intentional or not [2], of toxins of animal origin, such as those secreted by the glands of some animals, such as toads. In particular, the parotid glands of the Bufo toad secrete several active molecules, such as bufadienolides, with toxic effects (hemolytic, cardiotoxic and cytotoxic activity) [3]. Bufadienolid poisoning represents a critical case due to its high potency (lethal doses in the decigrams/kg range [4]) and its ability to cross the blood-brain barrier and inhibit P-glycoprotein-mediated efflux, concentrating this toxin in tissues including the central nervous system [5]. In general, bufadienolides have effects similar to those of cardiac glycosides, inhibiting the sodium potassium adenosine triphosphatase pump [6] and altering sodium, potassium and calcium channels. This effects lead to arrhythmias and central nervous system stimulation [7]. Ingestion of toad soup or eggs has resulted in death and severe intoxication [8]. In general, these poisonings respond well to treatment with digoxin specific antibody fragments. Due to the rarity of these poisonings in humans in Central Europe (dogs are often the main victims) [9], their identification is often difficult and common symptoms are often treated with atropine, digoxin inhibitors and gastric lavage as a precaution. The following is a case report of a child poisoned by toad eggs in Italy, in which a method of toxin identification using liquid chromatography (LC) coupled with tandem mass spectrometry (MS/MS).

## 2 Case description

In May 2022, an 8-year-old autistic boy, after an accidental ingestion of water from a small stream, experienced nausea, dizziness, agitation and drowsiness after walking in a mountain stream in Tuscany. This is the time of year when toads lay their eggs and fertilise them, especially in shallow waters. Observing his son's vomit, the father noticed something spherical, similar to fish or toad eggs, and decided to collect them in a small bag. In the emergency room, the medical staff observed drowsiness, bradycardia (48–52), and junctional rhythm in the patient. The child was then transferred to the children's hospital, where gastric lavage and charcoal decontamination were not performed due to the child's lack of compliance. The patient presented with a normal chest and abdomen, valid but arrhythmic heart tones, heart rate 70 bpm, blood pressure 127/51 mmHg, 98 % saturation, potassium equal to 6.7 mEq/L. Initially, a blood sample from the child was tested for digoxin, which showed a concentration of 0.68 ng/ml. The child was transferred to the pediatric intensive care unit (PICU) because of persistent drowsiness and junctional rhythm. The presence of bufadienolides was tested in toad eggs promptly collected by the child's father near the river bathing area. The eggs were sent to the Laboratory of Occupational Hygiene and Environmental Toxicology of the University of Florence to determine the nature of the poisoning. A weighted amount of the sample was homogenized, added with methanol and centrifugated. The supernatant was thus recovered, dried and resuspended in a water 0.1 % formic acid solution. The extract was analysed by high performance liquid chromatography (HPLC) triple quadrupole MS, Alliance e2695-Quattro Micro API (Waters, USA), using fragment ions generated by collision induced fragmentation. The chromatographic analysis was performed by a YMCPack ODS-AQ 5 μm, 2.1 × 250 mm column (Cat. No. AQ12S05-25Q1QT, prod. YMC Co. LTD, Japan) using a solution of water, 0.1 % formic acid and 60 % acetonitrile/40 % methanol as mobile phases. The flow rate was 0.3 ml/min and the column temperature was 25 °C; 20 μl of extract was injected. The total run time was 43 min. MS was performed using multiple reaction monitoring (MRM) and positive electrospray ionization (ESI+). In the immediate absence of a specific toxin target and corresponding analytical standard, we carried out an investigation of the analytical data: having suspected toad egg poisoning, we carried out a literature search of the mass spectra and transitions of the major bufotoxins to investigate the analysis of the egg homogenate sample (Table 1). The *m*/*z* transitions of the most likely encountered bufadienolides were obtained from the literature [10,11].

**Table 1:** Investigated molecules and their relative *m*/*z* transitions for the LC-MS/MS analysis.

Molecule	Transition	Collision energy
Bufalin	387.2 → 255.1	25.0
	387.2 → 369.6	
Arenobufagin	417.3 → 399.3(quantifier)	20.0
	417.3 → 371.3(qualifier)	
Cinobufagin	443.2 → 365.1	20.0
	443.2 → 347.4	
Resibufogenin	385.2 → 367.1	20.0
	385.2 → 349.2	

**Fig. 1:**
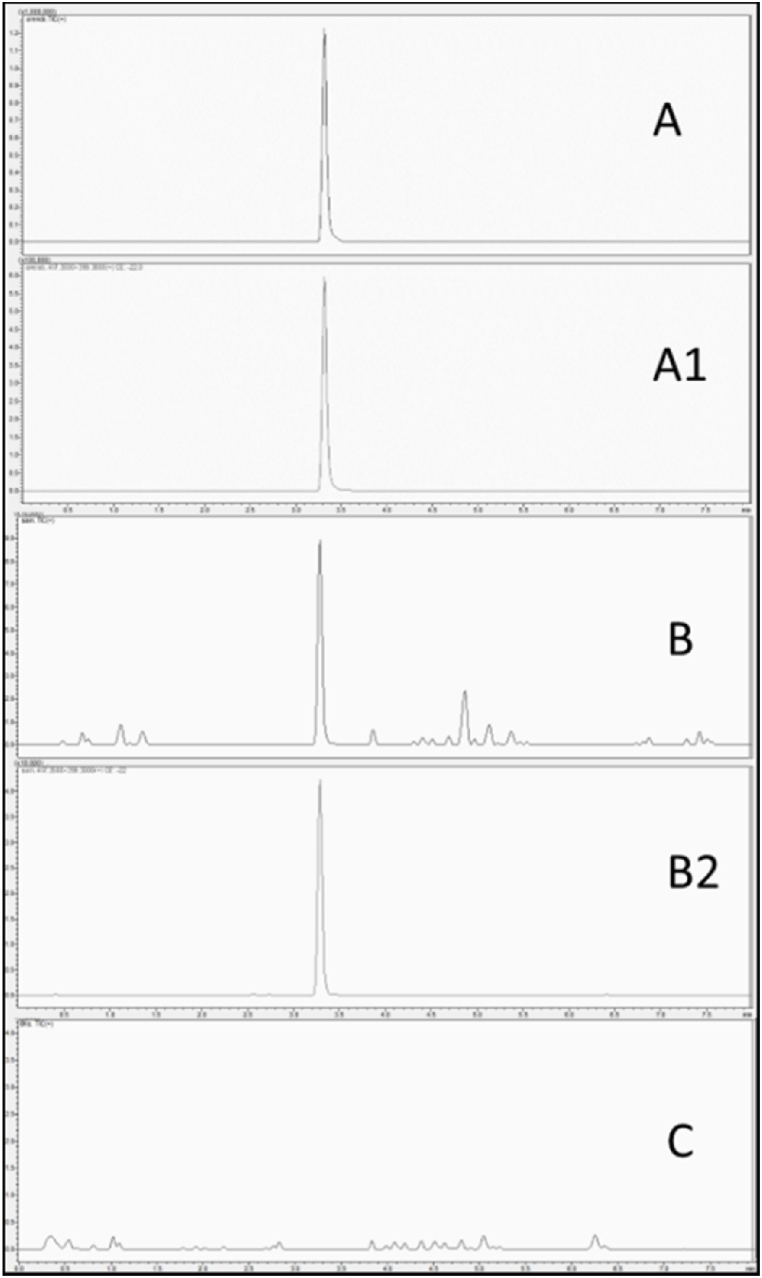
Chromatogram obtained for Arenobufagin (retention time 3.47 min): A) Analytical standard Total Ion Count (TIC) and A1) 417.3 → 399.3 transition, B) sample of toad eggs TIC and B1) 417.3 → 399.3 transition, C) Blank serum TIC.

Preliminary results showed the presence of a significant chromatographic signal with the transitions that could be associated with arenobufagin. This indication was communicated to the clinicians, who treated the patient with a digoxin-specific anti-body, atropine and insulin to counteract the atrioventricular dissociation and correct the hyperkalemia. After 20 minutes, the heart rhythm became sinusoidal and the blood potassium level was restored to physiological levels. Finally, the patient was discharged as there were no further abnormal heart rhythms. After 4 days, thanks to the standard purchased from the laboratory, the bufalin-like substance present in the toad egg homogenate was identified as arenobufagin thanks to the retention time and MRM signal obtained by LC-MS/MS analysis in MRM mode of the standard. The analyte was quantified at 0.021 ng/mg of eggs (Fig. 1).

Finally, the thawed serum of the child was analysed, but no bufadeinolides were found, probably due to the very low concentration of it and the modification of the matrix by the repeated thawing cycle used for the previous analyses.

## 3 Discussion

Over 200 species of Bufo toads are known in the world. Historically, these toads have been used in religious and healing rituals. All Bufo species produce biologically active substances, such as dopamine, epinephrine, norepinephrine, serotonin, bufotenine, bufagenins, bufotoxins and indolealkylamines, but there is variation in the amount of each substance produced by different toads. Toad poisoning is a significant problem in pets (dogs and cats). The case presented in this study is an accidental human poisoning by ingestion of toad eggs. Human cases of poisoning are extremely rare, but ingestion of toad egg soup has resulted in significant toxicity in certain cultures. With this in mind, an analytical method was developed and tested to provide clinicians with useful information on rare toxins such as bufadeinolides. Thanks to LC/MS-MS, an analytical platform with proven efficiency in pharmacotoxicology [12,13], and the use of MRM based on a large number of spectra from the literature, the substance present in the toad egg sample collected from the accident site was identified as arenobufagin. This qualitative information was used in this case to confirm the presence of the toxic toad at the spill site. Due to the time required to set up the analytical method and purchase the analytical standard, the clinicians had already treated the patient with digoxin-specific antibodies, however, having in a specialised laboratory unit such a versatile approach to poisoning, even if not analytically validated, could provide useful additional information.

## 4 Conclusions

Poisoning can be a challenge for clinicians: the introduction of mass spectrometry into the highly automated clinical laboratory is a powerful solution. Modern instrumentation and mass spectrometry databases provide useful information on various xenobiotics, with the advantages of using small sample volumes but the need for specialised personnel.

## Funding

This research did not receive any specific grant from funding agencies in the public, commercial, or not-for-profit sectors.

## CRediT authorship contribution statement

**Alessandro Bonari:** Writing – original draft, Data curation, Conceptualization. **Mauro Leucio Mattei:** Formal analysis, Data curation. **Giovanni Cappelli:** Writing – original draft, Visualization, Formal analysis. **Francesca Romano:** Data curation, Conceptualization. **Nicoletta Cini:** Writing – review & editing, Supervision. **Francesca Luceri:** Data curation. **Donato Squillaci:** Writing – review & editing. **Stefano Dugheri:** Methodology. **Alessandra Fanelli:** Supervision. **Nicola Mucci:** Supervision.

## Declaration of competing interest

Alessandro Bonari: None.

Mauro Leucio Mattei: None.

Giovanni Cappelli: None.

Francesca Romano: None.

Nicoletta Cini: None.

Francesca Luceri: None.

Donato Squillaci: None.

Stefano Dugheri: None.

Alessandra Fanelli: None.

Nicola Mucci: None.

## Data Availability

No data was used for the research described in the article.
